# Histological and Immunohistochemical Characterization of Vomeronasal Organ Aging in Mice

**DOI:** 10.3390/ani11051211

**Published:** 2021-04-22

**Authors:** Violaine Mechin, Patrick Pageat, Eva Teruel, Pietro Asproni

**Affiliations:** 1 Tissue Biology and Chemical Communication Department, IRSEA, Institute of Research in Semiochemistry and Applied Ethology, 84400 Apt, France; p.asproni@group-irsea.com; 2 Research and Education Board, IRSEA, Institute of Research in Semiochemistry and Applied Ethology, 84400 Apt, France; p.pageat@group-irsea.com; 3 Statistics and Data Management Service, IRSEA, Institute of Research in Semiochemistry and Applied Ethology, 84400 Apt, France; e.teruel@group-irsea.com

**Keywords:** vomeronasal organ, aging, pathology, histology, chemoreception

## Abstract

**Simple Summary:**

Chemical communication has been intensely studied and the importance of its role in animal life has been ascertained. Located in the nasal cavity, the vomeronasal organ is one of the main actors in charge of chemical reception. Alterations of this organ have proven to modify behavioral responses to semiochemical expositions. For all the other organs, a well-known origin of alteration is aging. The objective of this study was to analyze this effect on the vomeronasal organ condition and to determine the nature of these potential changes. This study demonstrates that this organ is significantly impacted by aging. In particular, old mice present strong signs of neuronal degeneration compared to adults.

**Abstract:**

The vomeronasal organ (VNO) plays a crucial role in animal behavior since it is responsible for semiochemical detection and, thus, for intra- and interspecific chemical communication, through the vomeronasal sensory epithelium (VNSE), composed of bipolar sensory neurons. This study aimed to explore a well-recognized cause of neuronal degeneration, only rarely explored in this organ: aging. Murine VNOs were evaluated according to 3 age groups (3, 10, and 24 months) by histology to assess VNSE changes such as cellular degeneration or glycogen accumulation and by immunohistochemistry to explore nervous configuration, proliferation capability, and apoptosis with the expression of olfactory marker protein (OMP), Gαi2, Gαo, Ki-67, and cleaved caspase-3 proteins. These markers were quantified as percentages of positive signal in the VNSE and statistical analyses were performed. Cellular degeneration increased with age (*p* < 0.0001) as well as glycogen accumulation (*p* < 0.0001), Gαo expression (*p* < 0.0001), and the number of cleaved-caspase3 positive cells (*p* = 0.0425), while OMP and Gαi2 expressions decreased with age (*p* = 0.0436 and *p* < 0.0001, respectively). Ki67-positive cells were reduced, even if this difference was not statistically significant (*p* = 0.9105). Due to the crucial role of VNO in animal life, this study opens the door to interesting perspectives about chemical communication efficiency in aging animals.

## 1. Introduction

The Jacobson organ, or vomeronasal organ (VNO), is a tubular structure bilaterally located in the nasal septum of most animals, such as amphibians, reptiles, and mammalians [1,2,3,4]. It is composed of a vomeronasal sensory epithelium (VNSE) and a non-sensory epithelium (NSE) arranged around a lumen [3,4]. Animals possess a variety of vomeronasal receptors, belonging to three families: formyl peptide receptors (FPRs) [5], vomeronasal type 1 receptors (V1R), and vomeronasal type 2 receptors (V2R) [6]. Vomeronasal receptors are the ones implicated in pheromone reception and thus in chemosensory responses [7,8,9]. V1R and V2R are respectively coupled to the Gαi2 and Gαo proteins and assure the function of semiochemical reception to the VNO [6,10,11]. These receptors detect semiochemicals in the vomeronasal lumen and transmit the message to the accessory olfactory bulb (AOB), which enables the animal to adapt its response to the signal. Thanks to this function, the VNO plays a vital role in chemical cues reception, in animal behavior and, thus, in its life [12,13,14].

Previous studies have shown that an experimentally damaged VNO could not assure a normal chemical communication among conspecifics, contributing to the onset of deficits in maternal, sexual, and social behaviors [15,16,17,18,19]. Moreover, spontaneous VNO alterations have been shown to be able to influence behavior in cats and were correlated with intraspecific aggression [20]. A study on pigs showed that vomeronasalitis was also associated with an increase in social conflicts depending on the VNSE inflammation intensity.

Many research studies have been conducted on aging in the central and peripheral nervous system, showing its evident impact on the brain and neuronal cell degeneration [21,22]. However, until today, only the influence of age on cell proliferation has been investigated in the VNO, showing that the cell number and epithelium density increased until 4 months, decreased between 4 and 8 months, and remained unchanged after [23,24]. An interesting study of Brann and colleagues showed the high capacity of the neuronal tissue of VNO reconstitution after a clinical injury, even in aged subjects, suggesting the preservation of dynamic stem cells during lifetime [25]. However, to the best of our knowledge, the nature of aging effects on the VNO neurons in normal conditions has not been already described. 

Therefore, this study aimed to characterize the biologic effects of aging on vomeronasal organ degeneration, in particular, in its sensorial part, as it is one of the most important tools for animal communication.

To achieve this purpose, the VNSE condition was assessed by histochemical and immunohistochemical approaches on three age groups of mice. The olfactory marker protein (OMP) was explored since it is commonly used as a marker of olfactory and vomeronasal neurons [26,27]. Gαi2 and Gαo proteins were studied to characterize the vomeronasal neuron type (expressing V1R or V2R) [6,10,11]. Finally, IHC markers of apoptosis and proliferation were also investigated between age groups to explore cellular turnover in the aging VNSE.

## 2. Materials and Methods

This study was carried out in strict accordance with the requirements of French and European Law on the protection of animals used for scientific purposes (2010/63/EU). The protocol was approved by the Committee C2EA125 on the Ethics of Animal Experiments of IRSEA (approval number: CE_2019_06_POVN).

### 2.1. Animals and Sampling Procedure

This study was conducted on the VNOs from 41 mice (82 VNSEs) *Mus musculus* (provided by Janvier Lab, Le Genest-Saint-Isle, France), split into three age groups, according to Flurkey’s classification [28]: G1: *n* = 11 mice 3 months old (adult), G2: 10 mice 10 months old (middle aged), G3: 20 mice 24 months old (old). Males and females were used in each group, belonging to two strains: the C57/6JRj (inbred) and the RjOrl:SWISS (outbred), to evaluate VNO changes in two commonly used genetic profiles. The animals’ repartition is described in Table S1 in Supplementary files.

Animals were maintained in the IRSEA’s facilities, at a temperature of 22 ± 2 °C and 60 ± 20% humidity with a 12–12 h light/dark cycle, with food and water ad libitum. At the required age, they were humanely euthanized with an intraperitoneal injection of Dolethal^®^ (100 mg/kg, Vetoquinol, Lure, France). The mice were then decapitated; their heads were immediately sampled and placed in a 10% formalin solution (pH 7.4) for one week to fix the tissues. Decalcification was performed by 48 h immersion in decalcifying solution (DDK, Milan, Italy). The noses were cut transversally to obtain 2–3 mm thin sections containing vomeronasal organ portions, processed, and paraffin-embedded according to routine histological methods. Sections of 3.5 µm thickness were made and dried overnight at 37 °C on SuperFrostPlus™ (Cat No. 10149870, Thermo Fisher Scientific, Illkirch, France) before being submitted to histological and immunohistochemical analyses.

### 2.2. Histochemical Analyses

Hematoxylin and eosin staining (H&E, BioOptica, Milan, Italy) was performed to assess the general condition of the VNO. Each VNSE was blindly classified on a scale score according to the degree of neuronal degeneration as 0 = nonexistent, 1 = weak, 2 = moderate, and 3 = strong. A score of zero was attributed when no physical signs of degeneration and no aberrant cellular vacuolization was detected. If any initial signs of degeneration were present, such as diffused vacuolization or non-homogenous cellular repartition, the tissue was noted as weak, with a score of 1. A moderate reduction of tissue density and diffuse vacuolization were qualified as score of 2. Finally, the maximal score of 3 was attributed in case of strong diffuse signs of degeneration, reduction of tissue density, and strong over vacuolization.

Periodic acid Schiff (PAS) staining (Cat No. C062AA, DiaPath SpA, Martinengo, Italy) was also performed following the manufacturer’s instructions to reveal glycogen accumulations. The images were analyzed with ImageJ software, using the “Color deconvolution” plug-In and the “H-PAS” vector. Red-purple color corresponding to the positive PAS staining and the VNSE surface were selected to obtain a percentage of PAS-positive areas in the selected VNSE surface.

### 2.3. Immunohistochemical Analyses

IHC was performed to evaluate the presence of proteins involved in VNO functionality and in cellular turnover.

After rehydration, tissues were pretreated with a microwave in 0.1 M citrate buffer pH 6 (Cat No. F/T0050, DiaPath SpA, Martinengo, Italy), 3 min and 30 s at 560 W, and 15 min at 210 W. Slides were then incubated with a peroxidase blocking solution (Cat No. ACA500, Scytek, Logan, UT, USA) for 30 min protected from light, followed by an UltraVision Protein Block incubation step (Cat No. TA-125-PBQ, Thermo Scientific, Carlsbad, CA, USA), for 10 min. The sections were then incubated for 1 h at room temperature with the primary antibodies anti-olfactory marker protein, anti-Gαo, anti-Gαi2, anti-cleaved caspase 3, or anti-Ki67, as described in Table 1.

After washing in TBS Tween 1%, a 10 min incubation was performed with a secondary biotinylated anti-species (UltraTek Anti-rabbit, Cat No. T/ABE125, or Anti-goat, Cat No. F/AGL125, ScyTek Laboratories, Logan, UT, USA), followed by a 10 min streptavidin-peroxidase incubation (Cat No: 12694067, Invitrogen, Carlsbad, CA, USA). Visualization was obtained with 3,3′ diaminobenzidine tetrahydrochloride (ImmPACT^®^ DAB Peroxidase Substrate, Cat No. SK4105, Vector Laboratories, Burlingame, CA, USA) and a hematoxylin counterstain. The sections were finally dehydrated through alcohols, cleared in xylene, and mounted. For each antibody, as negative controls, the primary antibodies were replaced with a nonimmune rabbit or goat serum, while for positive controls for Ki-67 and cleaved caspase 3, murine lymph node sections were included in each IHC run. 

The software ImageJ was used to measure IHC positivity of each antibody in the VNSEs.

To quantify the OMP and G proteins, the plugin “Color deconvolution” was used in Image J to measure the positive pixels as it allows for extrapolation of the DAB staining in the manually selected VNSE area. The software automatically calculated the percentage of positivity on the VNSE surface.

For Ki-67 and cleaved caspase 3, positive cells were manually counted on each section using the ImageJ plugin “cell counter”, then the percentage of positive cells among the total VNSE cells was calculated. IHC quantification methods for each antibody are presented in Table 2.

### 2.4. Statistical Analyses

Data analyses used SAS 9.4 software (Copyright© 2021–2012 by SAS Institute Inc., Cary, NC, USA). The significance threshold was classically fixed at 5%. The principal explanatory variable was the age of the mice (3, 10, or 24 months). Sex (M/F) and strains (inbred/outbred) were also collected to see if the effect of age on the VNO degeneration was different depending on the sex and/or strains. For that, age * sex and age * strains interactions were included in the models in addition to age, sex, and strains factors. Two VNSEs (from left and right parts of each VNO) were available from each mouse. Thus, when possible, each mouse was considered as a random factor in the models.

For OMP expression, accumulated glycogen and Gαo protein, a general linear mixed model was applied. The assumptions of residual normality and homoscedasticity were verified. Post hoc multiple comparisons were studied using the Tukey–Kramer adjustment. 

For the Gαi2 protein, assumptions of normality and homoscedasticity were not verified. Other distributions were tested in order to try to apply a generalized linear mixed model, but no distribution matched with this variable. Additionally, data transformation did not lead to validation of the conditions of application. As a result, only an age effect was studied using the nonparametric test of Kruskal–Wallis. Wilcoxon Mann–Whitney tests were performed to compare age groups two by two. The Bonferroni correction was applied considering the multiplicity of the tests.

For cellular degeneration (non-existent, weak, moderate, strong), it was not possible to include the mice as a random effect because the results were unstable using a multinomial mixed model. Consequently, a classical ordinal multinomial model was performed using the LOGISTIC procedure. Odds ratios were computed for multiple comparisons. 

For proliferation and apoptosis parameters, which were count data, mixed Poisson models were applied. The total number of cells was added to the models as an offset term to take into account the fact that this number was not the same for all VNOs studied. The dispersion of the models was studied by regarding the Pearson chi-square/DF statistic. Post hoc multiple comparisons were studied using the Tukey–Kramer adjustment.

## 3. Results

For each parameter, no statistical differences were found comparing sex, strains, and sex * age or strain * age interactions. Statistical differences were only found when ages were compared.

Descriptive data are shown in Table 3.

### 3.1. Cellular Degeneration

**Figure 1:**
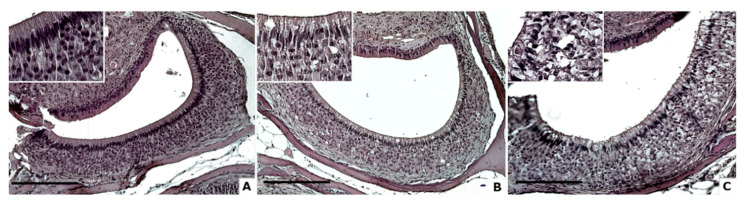
Age effect on cellular degeneration in mouse VNSEs (**A**–**C**). H&E staining was used to highlight histological degeneration signs, such as vacuolization or structure alterations (Inserts: ×40 magnification of VNSE samples) at 3 months (**A**), 10 months (**B**), and 24 months (**C**). (Objective × 20, Scale bar = 200 µm).

Qualitative scores of cellular degenerations in VNSEs were studied according to age groups. A significant effect of age was revealed (DF = 2; X^2^ = 18.7; *p* < 0.0001; multinomial model). A post hoc comparison showed significant differences between 3 and 24 months (odds ratio (OR) = 0.17; *p* < 0.0001) and between 10 and 24 months (OR = 11.5; *p* = 0.005). The difference observed between the mice at ages 3 and 10 months was not significant (OR = 1.9; *p* = 0.3174) (Figure 1).

### 3.2. Increased Quantity of Accumulated Glycogen with Age

**Figure 2:**
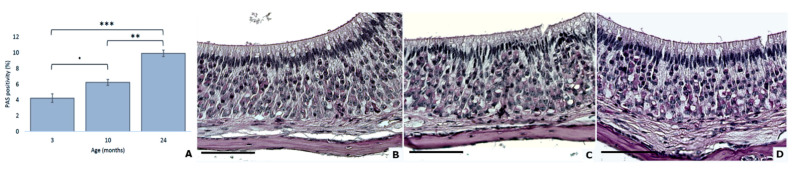
Age effect on PAS positivity in mouse VNSEs (**A**). Glycogen presence in mouse VNSEs aged 3, 10, and 24 months with PAS staining. Data are shown as the mean ± SD, * *p* ≤ 0.1; ** *p* ≤ 0.01; *** *p* ≤ 0.001 as indicated (**B**–**D**). PAS staining was used to stain the glycogen accumulations a red-purple color at 3 months (**B**), 10 months (**C**), and 24 months (**D**). Pathologic glycogen accumulation was often located in the vacuoles in the VNSEs of 24-month-old mice. (Objective × 40, Scale bar = 100 µm).

Accumulated glycogen was quantified with PAS staining, and significant variations were observed among the age groups (DF = 2; F = 23.30; *p* < 0.0001; general linear mixed model). The statistical model indicated that the observed difference showed increasing PAS positivity between 3 months and 24 months (*p* < 0.0001) and between 10 months and 24 months (*p* = 0.0004) (Figure 2).

### 3.3. OMP Expression Decreases with Age

**Figure 3:**
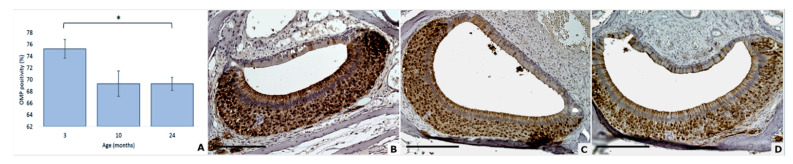
Age effect on olfactory marker protein positivity in mouse VNSEs (**A**). OMP expression in mouse VNSEs aged 3, 10, and 24 months with immunohistochemical staining of the OMP protein. Data are shown as the mean ± SD, * *p* ≤ 0.05 (**B**–**D**). Immunohistochemical staining of OMP with a DAB chromogen and hematoxylin counterstain at 3 months (**B**), 10 months (**C**), and 24 months (**D**). (Objective × 20, Scale bar = 200 µm).

Regarding the presence of olfactory marker protein in the VNSE of mice, statistical analysis showed that age significantly impacted the expression of this protein (DF = 2; F = 3.84; *p* = 0.0436; general linear mixed model). Post hoc multiple comparisons revealed a significant difference between 3 and 24 months (*p* = 0.0418). No differences were observed between 3 and 10 months (*p* = 0.2087) or between 10 and 24 months (*p* = 0.9998) (Figure 3).

### 3.4. Gαi2 and Gαo Proteins Modifications

Statistical analysis showed that the expressions of the Gαi2 and Gαo proteins are both highly dependent on aging, in an opposite manner. Indeed, the expression of Gαi2 protein decreased with age (DF = 2; Ki^2^ = 37.47; *p* < 0.0001; Kruskal–Wallis test) while the expression of Gαo protein was increased (DF = 2; F = 58.68; *p* < 0.0001; general linear mixed model). 

**Figure 4:**
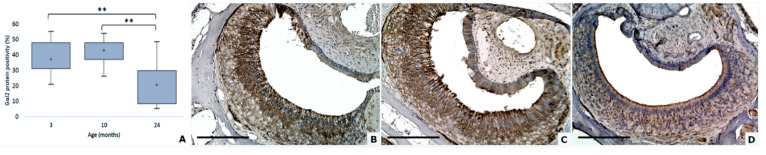
Gαi2 protein expression decreases with aging (**A**). Gαi2 expression in mouse VNSEs aged 3, 10, and 24 months with immunohistochemical staining of the Gαi2 protein. Data are shown as the median, ** *p* ≤ 0.01 (**B**–**D**). Immunohistochemical staining of Gαi2 with a DAB chromogen and hematoxylin counterstain at 3 months (**B**), 10 months (**C**), and 24 months (**D**). (Objective × 20, Scale bar = 200 µm).

Wilcoxon tests indicate that Gαi2 was significantly higher in the 3 months than the 24 months group (*p* = 0.0003) and higher in the 10 months group than the 24 months group (*p* = 0.0003) (Figure 4).

**Figure 5:**
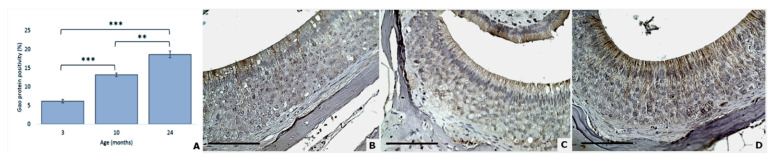
Gαo protein expression increases with aging (**A**). Gαo expression in mouse VNSEs aged 3, 10, and 24 months with immunohistochemical staining of the Gαo protein. Data are shown as the mean ± SD, ** *p* ≤ 0.01; *** *p* ≤ 0.001 as indicated (**B**–**D**). Immunohistochemical staining of Gαo with a DAB chromogen and hematoxylin counterstain at 3 months (**B**), 10 months (**C**), and 24 months (**D**). (Objective × 40, Scale bar = 100 µm).

For Gαo, significant differences were revealed between 3 and 10 months (*p* < 0.0001), 3 and 24 months (*p* < 0.0001), and 10 and 24 months (*p* = 0.0048) (Figure 5).

### 3.5. Proliferation and Apoptosis Factors

**Figure 6:**
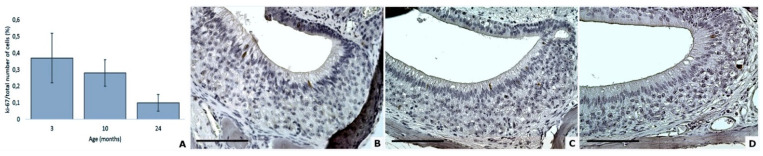
Aging has no significant effects on Ki67 protein expression in mouse VNSEs (**A**). Proliferative marker in mouse VNSEs aged 3, 10, and 24 months obtained with immunohistochemical staining of the Ki67 protein. Data are shown as the mean ± SD (**B**–**D**). Immunohistochemical staining of Ki67 with a DAB chromogen and hematoxylin counterstain at 3 months (**B**), 10 months (**C**), and 24 months (**D**). (Objective × 40, Scale bar = 100 µm).

Age did not significantly impact the expression of Ki67 protein in the VNSE of aging mice (DF = 2; F = 0.09; *p* = 0.9105; Poisson model) (Figure 6).

**Figure 7:**
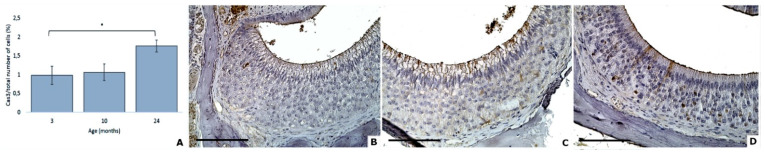
Age effects on the apoptosis of mouse VNSEs (**A**). Cleaved caspase 3 expression in mouse VNSEs aged 3, 10, and 24 months obtained with immunohistochemical staining of this protein. Data are shown as the mean ± SD, * *p* ≤ 0.1 (**B**–**D**). Immunohistochemical staining of cleaved caspase 3 was used to reveal the protein with a DAB chromogen and hematoxylin counterstain at 3 months (**B**), 10 months (**C**), and 24 months (**D**). (Objective × 40, Scale bar = 100 µm).

On the converse, age had an impact on the expression of cleaved caspase 3 protein (DF = 2; F = 3.42; *p* = 0.0425; Poisson model). Multiple comparisons informed us that a difference was obtained thanks to a trend between 3 and 24 months (*p* = 0.0748). No differences were observed between 3- and 10-months groups (*p* = 0.9688) or between 10- and 24-months groups (*p* = 0.1538) (Figure 7).

For each parameter, sex and strain variables were statistically analyzed, and their interactions with aging were verified. No interactions were found in all cases, and no differences were observed according to these variables.

## 4. Discussion

Our results showed that aging influences the vomeronasal organ structure. Moreover, this study allowed us to characterize the engendered effects and their delays in appearance.

Our results indicated that the cellular degeneration seems to appear after the age of 10 months in mice, which is considered middle age in this species. In fact, our data demonstrated an increase of the degenerative changes with aging, between 3 and 24 months, and between 10 and 24 months. Previous studies [22,23] observed an increase of the VNO volume until the 4 months of age, followed by a decrease stabilized around the 8 months. Even if our study evaluated other kinds of alterations and involved only the VNSE, it seems possible to suggest that around 8–10 months of age, the aging process begins to alter the VNO’s structure. The observed vacuoles may result from water or lipid lysosomal accumulation, and these changes are frequently due to defective cellular metabolism [29]. It is well known that the histological sections can present artefacts that could influence the analysis of some degenerative signs. However, to reduce and to homogenize this risk, all the samples were submitted to the same processing procedure.

The metabolism efficiency was also tested with PAS staining, a technique that allows the staining of glycogen, which is commonly present in low quantities in the nervous system to assure the vital energy required by neuronal activation [30,31]. An aberrant accumulation of this glucosidal polymer in neurons can drive early aging and neuronal degeneration and is frequently found in neurodegenerative diseases, such as Alzheimer, Parkinson, and dementia diseases [32,33]. In this study, glycogen accumulation appears from the age of 10 months, which could impact the neurons’ function, and, consequently, VNO function. Moreover, PAS positive staining was often located in the neurons’ vacuoles, suggesting a possible alteration of the cellular metabolism, as commonly occurs during normal aging [34,35].

As showed by the statistical analysis, olfactory marker protein was slightly but significantly decreased between adults and old mice. Since this protein is expressed by vomeronasal neurons, this finding could be due to the cellular degeneration revealed by the VNSE histological analysis, or to an age influence on the cellular protein synthesis and accumulation. OMP has a crucial role in neuronal functionality and nervous signal transmission [36], and thus a reduction of this protein could also suggest a VNO dysfunction. However, our results showed only a weak statistical difference, therefore, their interpretation should be taken with prudence. Moreover, further investigations are needed to deeper explore these changes and their possible implications.

The Gαi2 protein, co-expressed on V1Rs in charge of the reception of volatile and steroidal molecules, has a major role in communication, and in maternal, sexual, and social pheromones’ detection [10,37,38]. Previous studies showed that when this protein was deleted or its expression decreased, the chemosensory behavior of animals was altered, leading to an increase of aggressive behaviors, and modified sexual behaviors [16,39,40]. This study reported that Gαi2 positivity was significantly decreased in the VNSE of aging mice. According to the previous literature, this phenomenon could lead to a reduction of the mouse’s communication capabilities, and thus to an increase in behavior disorders, such as aggression.

The Gαo protein is co-expressed on V2Rs, the receptors of nonvolatile peptides, large proteins implicated in territorial marking and alarm pheromones, and plays a key role in social behavior [40]. Our statistical analysis seems to indicate a strong increase of this protein in VNSEs according to age, in contrary to the Gαi2 results. Since these data were quite surprising compared to the trends of the other markers included in this study, the authors’ opinion is that a compensatory mechanism can be presumed, as already proposed in the literature for other G proteins [41]. However, it is well known that V2Rs co-express the MHC class 1b molecules, in particular, the M10 family, which is linked to β2microglobuline (β2m) protein [42]. This protein significantly increases according to age in another murine peripheral sensory epithelium, such as the retina, as well as in various brain regions [43]. These findings could partially explain our observation on Gαo increase in old mice VNSE. Interestingly, the IHC stain of our samples was less intense than that observed in previous studies [44], probably because our protocol used a lower dilution, in order to obtain quantitative differences among samples, and not to assess the positivity or the negativity of the tissues. Consequently, this weak signal does not allow to exactly quantify the ratio between the two G-proteins in old mice. Finally, also considering the low percentages, these results should be interpreted with caution and corroborated with further studies.

To explore if cellular turnover is maintained during aging, and if its alteration could be a factor in the onset of degenerative phenomena, we also investigated apoptotic and proliferative cellular activities, as some studies have already demonstrated that neuronal regeneration is preserved after surgical injuries during aging in mice [25]. Our results showed that the apoptotic marker was indeed higher in aged mice. The expression of proliferative protein seems to be decreased with aging, but no significant effect was revealed. Even if these results are not strong enough to conclude about the cellular turnover, they may suggest that the number of dead cells is not balanced by the proliferation of new cells, indicating a possible decrease of the regeneration capabilities of the aging VNSE, which could be one of the causes of the degenerative phenomena described in this article. The study of Brann and Firestein [25] showed that the VNSE proliferative activity remains robust in response to an experimental lesion even in 24 months-old mice, while this activity is slightly lower in the non-lesioned old VNO, suggesting that the increased proliferation is caused by the induced reparative process. 

Since the VNO shares some important characteristics with the olfactory mucosa [8], in particular, the histological pattern of the VNSE, it is interesting to compare our results to those reported in literature on the aging of the olfactory epithelium. Conversely to the VNO, the aging of the olfactory mucosa has been largely studied, in particular, in mice and humans. The most frequent changes observed in aging are the decrease of the thickness of the epithelium with a concurrent localized metaplasia, pigments deposits, and an important reduction of the neuroepithelium vascularization [45,46]. In our study, the main histological finding was the diffuse degeneration of the VNSE, which is less reported in olfactory neuroepithelium. Interestingly, among the causes that can lead to the aging of the olfactory mucosa, the decrease of the mitotic activity seems to play a crucial role, as well as the increase of the ratio of dead or dying neurons to the number of live cells [46]. As previously mentioned, our results do not allow to draw firm conclusion on cellular turnover in the aged VNSE, even if a dis-balance before newborn and apoptotic cells can be presumed.

This study permits us to affirm that aging has a real impact on the neuronal cells of the VNO. Due to the role of this organ and of these sensory neurons, these changes may impact the mouse’s chemical communication capabilities, and, consequently, the animals’ behavior. Indeed, neuron functionality seems to be altered, as shown by the G proteins modification in natural conditions. However, these hypotheses should be confirmed by further studies evaluating the functionality of the VNO in old animals and their behavioral responses to semiochemicals’ stimuli.

Considering the crucial role of the VNO in animals’ communication, these effects are supposed to directly impact also animals’ behavior and consequently, their life. To confirm these hypotheses, further studies are needed to investigate the age effect on the behavioral response after the exposition to semiochemical stimuli. Other investigations should be also led on other mammals in order to explore VNO aging and its impact in domestic and farm animals, to obtain more knowledge about their capabilities to communicate, and to adapt to their environment throughout the aging process.

## 5. Conclusions

In this study, we showed that aging has a strong impact on the neuronal cells of the vomeronasal organ, modifying their structure and their metabolism. Further studies are needed to evaluate if these changes may influence chemical communication capabilities in old animals and, consequently, to induce behavioral changes. To the best of our knowledge, this study is the first to characterize the effects of aging on the vomeronasal neuronal cells of the mouse and in animals in general.

## Figures and Tables

**Table 1:** List of antibodies used in this study.

Antibody	Host	Clonality	Dilution	Reference	Supplier
Anti-Olfactory Marker Protein (OMP)	Rabbit	Polyclonal	1:10,000	O7889	Sigma, Saint-Louis, MO, USA
Anti-Gαi2 protein	Rabbit	Monoclonal	1:200	Ab157204	Abcam, Cambridge, UK
Anti-Gαo protein	Goat	Polyclonal	1:200	Sc26769	Santa Cruz Biotechnologies, CA, USA
Anti-Cleaved Caspase 3	Rabbit	Polyclonal	1:1000	9661	Cell Signaling Technology, Danvers, MA, USA
Anti-Ki-67	Rabbit	Monoclonal	1:50	MA514520	Life Technologies, Carlsbad, CA, USA

**Table 2:** Studied parameters and their quantification.

Antibody	Targeted Cells/Molecules	Quantification Method
Anti-Olfactory Marker protein (OMP)	Mature neurons quantification	% of positivity signal/VNSE surface
Anti-Gαi2 protein	V1R neurons	% of positivity signal/VNSE surface
Anti-Gαo protein	V2R neurons	% of positivity signal/VNSE surface
Anti-Cleaved Caspase 3	Apoptosis	Cell number/VNSE surface
Anti-Ki-67	Proliferation activity	Cell number/VNSE surface

**Table 3:** Distribution according to the age of the histochemical and immunohistochemical parameters.

Studied Parameters	3 Months (*n* = 20)	10 Months (*n* = 22)	24 Months (*n* = 40)
Cellular Degeneration	*n* (%)		
non-existent	2 (10.0)	5 (22.7)	0 (0.0)
weak	13 (65.0)	13 (59.1)	15 (37.5)
moderate	5 (25.0)	4 (18.2)	17 (42.5)
strong	0 (0.0)	0 (0.0)	8 (20.0)
	% area (stderr)		
PAS positivity	4.2 (0.5)	6.3 (0.4)	9.9 (0.4)
OMP positivity	75.3 (1.6)	69.3 (2.2)	69.3 (1.1)
Gαi2 positivity	39.1 (2.2)	42.7 (1.6)	20.8 (2.1)
Gαo positivity	6.1 (0.5)	13.2 (0.5)	18.6 (0.9)
	% positive cells (stderr)		
Proliferative cells	0.4 (0.2)	0.3 (0.1)	0.1 (0.1)
Apoptotic cells	1.0 (0.2)	1.1 (0.2)	1.8 (0.2)

## Data Availability

The data presented in this study are available within the article in the supplementary files.

## Supplementary Materials

The following are available online at https://www.mdpi.com/article/10.3390/ani11051211/s1, Table S1: Animals’ repartition and raw data.
